# Optimal timing of cholecystectomy after percutaneous gallbladder drainage for severe cholecystitis

**DOI:** 10.1186/s12876-017-0631-8

**Published:** 2017-05-31

**Authors:** Koetsu Inoue, Tatsuya Ueno, Orie Nishina, Daisuke Douchi, Kentaro Shima, Shinji Goto, Michinaga Takahashi, Chikashi Shibata, Hiroo Naito

**Affiliations:** 1 Department of surgery, South Miyagi Medical Center, 38-1 Aza-nishi, Ogawara, Shibata-gun, Miyagi 989-1253 Japan; 2 Division of Gastroenterological Surgery, Department of Surgery, Tohoku Medical and Pharmaceutical University Hospital, 1-12-1 Hukumuro, Miyagino-ku, Sendai, Miyagi Japan

**Keywords:** Cholecystitis, Percutaneous transhepatic gallbladder drainage, Cholecystectomy

## Abstract

**Background:**

The Tokyo guideline for acute cholecystitis recommended percutaneous transhepatic gallbladder drainage followed by cholecystectomy for severe acute cholecystitis, but the optimal timing for the subsequent cholecystectomy remains controversial.

**Methods:**

Sixty-seven patients who underwent either laparoscopic or open cholecystectomy after percutaneous transhepatic gallbladder drainage for severe acute cholecystitis were enrolled and divided into difficult cholecystectomy (group A) and non-difficult cholecystectomy (group B). Patients who had one of these conditions were placed in group A: 1) conversion from laparoscopic to open cholecystectomy; 2) subtotal cholecystectomy and/or mucoclasis; 3) necrotizing cholecystitis or pericholecystic abscess formation; 4) tight adhesions around the gallbladder neck; and 5) unsuccessfully treated using PTGBD. Preoperative characteristics and postoperative outcomes were analyzed.

**Results:**

The interval between percutaneous transhepatic gallbladder drainage and cholecystectomy in Group B was longer than that in Group A (631 h vs. 325 h; *p* = 0.031). Postoperative complications occurred more frequently when the interval was less than 216 h compared to when it was more than 216 h (35.7 vs. 7.6%; *p* = 0.006).

**Conclusions:**

Cholecystectomy for severe acute cholecystitis was technically difficult when performed within 216 h after percutaneous transhepatic gallbladder drainage.

**Electronic supplementary material:**

The online version of this article (doi:10.1186/s12876-017-0631-8) contains supplementary material, which is available to authorized users.

## Background

Acute cholecystitis (AC) is a common disease for which laparoscopic cholecystectomy (LC) has become the standard treatment [1]. However, in patients with severe AC, the rate of complications such as bile leak, common bile duct injury, and bowel injury is high after LC [2], suggesting an association between severity of inflammation and difficulty of LC. Therefore, evaluation of the severity of AC is important in determining the appropriate treatment.

The Tokyo Guidelines in 2007 was issued as the first international guidelines for the diagnosis and treatment of AC and has been recently revised as the Tokyo Guidelines 2013 (TG13) [3, 4]. The TG13 suggested that the criteria for AC should be based on clinical symptoms, physical examination, blood tests, and imaging findings and classified AC into three grades of inflammation: mild (grade I), moderate (grade II), and severe (grade III) (Table 1). The TG13 also recommended appropriate therapy depending on the grade of AC. Grade I patients are candidates for immediate LC; grade II patients could undergo either LC or percutaneous transhepatic gallbladder drainage (PTGBD); and grade III patients are strongly recommended to undergo immediate PTGBD.

**Table 1:** Severity classification of acute cholecystitis by the Tokyo guidelines 2013

Grade	Definition	
I (Mild)	Acute cholecystitis that does not meet the criteria for grade III or grade II cholecystitis	
	Acute cholecystitis in a healthy patient with no organ dysfunction. Inflammatory changes in the gallbladder are mild, making cholecystectomy a safe and low-risk procedure.	
II (Moderate)	Grade II acute cholecystitis is associated with any one of the following conditions	
	1	Elevated white blood cell count (>18,000/mm^3^)
	2	Palpable tender mass in the right upper abdominal quadrant
	3	Duration of complaints > 72 h
	4	Marked local inflammation (gangrenous cholecystitis, pericholecystic abscess, hepatic abscess, biliary peritonitis, and emphysematous cholecystitis)
III (Severe)	Grade III acute cholecystitis associated with dysfunction of any one of the following organs/systems	
	1	Cardiovascular dysfunction defined as hypotension requiring treatment with dopamine ≥ 5 μg/kg per min or any dose of norepinephrine
	2	Neurologic dysfunction defined as decreased level of consciousness
	3	Respiratory dysfunction defined as a PaO2/FiO2 ratio < 300
	4	Renal dysfunction defined as oliguria or creatinine > 2.0 mg/dl
	5	Hepatic dysfunction defined as PT-INR > 1.5
	6	Hematologic dysfunction defined as platelet count < 100,000/mm^3^

In the early 1980s, Radder introduced the procedure of PTGBD to immediately improve the symptoms of AC [5]. In some patients who undergo PTGBD and subsequent LC, technical difficulties and postoperative complications associated with severe fibrosis and gallbladder adhesion may be encountered [6]. Therefore, it is very important to determine the optimal timing of cholecystectomy after PTGBD; however, this is still controversial [4].

The aim of this study was to assess the effect of the interval between PTGBD and cholecystectomy on the technical difficulty of cholecystectomy and postoperative complications. We also aimed to determine the optimal timing of cholecystectomy after PTGBD.

## Methods

**Fig. 1:**
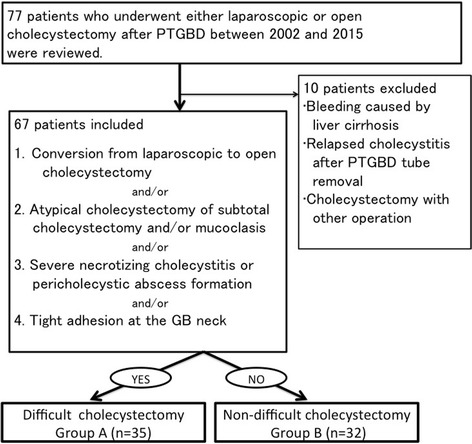
Study flow chart. PTGBD: percutaneous transhepatic gallbladder drainage

The medical records of the 77 study patients who underwent either laparoscopic or open cholecystectomy after PTGBD for AC between 2002 and 2015 were retrospectively reviewed (Fig. 1). Ten patients who met one of the conditions below were excluded from this study: 1) hemorrhage associated with liver cirrhosis, 2) relapsed cholecystitis after PTGBD tube removal, and 3) need for procedures other than cholecystectomy. At least one board-certified surgeon of the Japan Surgical Society participated in all surgeries.

Since there was no quantitative evaluation for the difficulty of cholecystectomy, we employed objective and subjective criteria in this study. Difficult cholecystectomy was defined as the presence of either one of these conditions: 1) conversion from laparoscopic to open cholecystectomy; 2) atypical cholecystectomy, such as subtotal cholecystectomy and/or mucoclasis; 3) necrotizing cholecystitis or pericholecystic abscess formation; 4) tight adhesions around the gallbladder neck; and 5) a patient unsuccessfully treated using PTGBD. In grade I and II cholecystites, timing of surgery following PTGBD was decided by each surgeon. In grade III cholecystitis, surgery was performed after recovery of patient from organ dysfunction. Decision of conversion during surgery depended on each surgeon. There were no definite criteria for performing either open cholecystectomy or laparoscopic cholecystectomy.

Necrotizing cholecystitis was confirmed by intraoperative and pathologic findings. The remaining 67 patients were divided into two groups according to the criteria mentioned above: the difficult cholecystectomy group (group A, *n* = 35) and the non-difficult cholecystectomy group (group B, *n* = 32). Preoperative characteristics and postoperative outcomes were analyzed. Detailed data of patient characteristics used in this study was shown in Additional file 1.

Data are shown as mean ± standard error mean (SEM). Categorical variables were analyzed by Chi-square test, whereas continuous data were analyzed by either two-tailed Student’s t test or Wilcoxon test, according to the result of the Shapiro–Wilk test. Statistical significance was defined as *P* < 0.05. The optimal cut-off value was calculated using a receiver operating characteristic (ROC) curve and was defined as the number that indicated the highest sum of the sensitivity and specificity on the ROC curve. Statistical analysis was performed with JMP Pro 11 software (SAS Institute, Cary, NC, U.S.A).

## Results

Among the 67 patients enrolled in this study, 41 (61.2%) were men, and the median age was 75.0 years. The severity of AC was grade I in 13 cases (19.4%), grade II in 42 cases (62.7%), and grade III in 12 cases (17.9%). There were 3 patients who were unsuccessfully treated using PTGBD (one patient: relapsed cholecystitis following PTGBD, 2 patients: bile peritonitis due to PTGBD), and rate of patients with unsuccessfully treated with PTGBD was 3.9%. Patients enrolled in this study, did not have troubles associated with PTGBD tube. Nine patients needed conversion from LC to open cholecystectomy, whereas 45 patients underwent LC. The reasons for conversion were as follows: 1) inadequate exposure of Calot’s triangle due to severe adhesion and inflammation in 8 cases and 2) accidental cystic duct injury in one case. In 13 patients, open cholecystectomy was selected at the beginning of the surgery. The reasons for selecting open cholecystectomy were as follows: 1) history of upper abdominal surgery in 6 cases, 2) Mirrizi syndrome at admission in 3 cases, 3) acute pancreatitis at admission in 2 cases, and 5) severe wall thickness in 2 cases.

The interval between PTGBD and cholecystectomy ranged from 2 to 4584 h, with the median at 360 h. Only seven cases had an interval between procedures of within 72 h. The detailed patient characteristics are shown in Table 2. The interval between PTGBD and cholecystectomy in Group B was significantly longer than that in Group A (325 ± 95.6 h vs. 631 ± 99.9 h; *p* = 0.031), whereas the interval between symptom onset and PTGBD did not differ between the two groups (77.5 ± 12.8 h vs. 78.0 ± 13.4 h; *p* = 0.978). All other parameters did not differ between the two groups. Table 3 shows that AST and γ-GTP were lower in group A than in group B (AST: 76.3 ± 38.6 IU/L vs. 196 ± 40.4 IU/L; *p* = 0.036 and γ-GTP: 95.3 ± 36.6 IU/L vs. 240 ± 38.3 IU/L; *p* = 0.008), but all other data including WBC and CRP did not differ between groups. Perioperative characteristics are shown in Table 4. Operating time (146 ± 5.8 min vs. 97.3 ± 6.1 min; *p* < .0001) and blood loss (180 ± 32.1 mL vs. 28.8 ± 33.6 mL; *p* = 0.0017) were significantly greater in Group A than in Group B. Distribution of surgical procedures in the 67 patients was as follows; Laparoscopic cholecystectomy: Group A, 21 cases (60.0%) and Group B, 24 cases (75.5%); Open cholecystectomy: Group A, 5 cases (14.3%) and Group B, 8 cases (25.0%); and Open conversion: Group A, 9 cases (25.7%) and Group B, 0 cases (0%). The rate of open cholecystectomy did not differ between the two groups (*p* = 0.268).

**Table 2:** Comparison of patient clinical characteristics according to the difficulty of cholecystitis

Variable	Group A	Group B	*P* Value
	(*n* = 35)	(*n* = 32)	
Age, years	69.3 ± 2.1	73.3 ± 2.2	0.192
Male sex, n (%)	22 (62.9)	19 (59.4)	0.770
BMI	24.3 ± 0.70	22.3 ± 0.73	0.053
Interval between onset and PTGBD, hours	77.5 ± 12.8	78.0 ± 13.4	0.978
Interval between PTGBD and cholecystectomy, hours	325.0 ± 95.6	631.0 ± 99.9	0.031
Fever on admission, n (%)			0.609
No	25 (71.4)	21 (65.6)	
Yes	10 (28.6)	11 (34.4)	
Abdominal pain on admission, n (%)			0.196
No	4 (11.4)	1 (3.1)	
Yes	31 (88.6)	31 (96.9)	
Tokyo Guidelines 2013			
Grade I	5 (14.3)	8 (25.0)	0.268
Grade II	22 (62.9)	20 (62.5)	0.976
Grade III	8 (22.9)	4 (12.5)	0.269
Hypertension	19 (54.3)	12 (37.5)	0.169
Diabetes mellitus	8 (22.9)	3 (9.4)	0.137

*BMI* Body mass index, *PTGBD* Percutaneous transhepatic gallbladder drainage

Continuous variables are presented as mean ± SEM

**Table 3:** Comparison of laboratory data on admission according to the difficulty of cholecystitis

Variable	Group A	Group B	*P* Value
	(*n* = 35)	(*n* = 32)	
White blood cells, 10^3^/μL	13.0 ± 0.99	14.6 ± 1.04	0.275
Hemoglobin, g/dL	13.2 ± 0.38	13.6 ± 0.40	0.474
Platelets, 10^3^/μL	181 ± 14.0	197 ± 14.6	0.429
CRP, mg/dL	13.2 ± 1.85	10.4 ± 1.93	0.284
Total bilirubin, mg/dL	1.6 ± 0.34	2.5 ± 0.35	0.053
AST, IU/L	76.3 ± 38.6	196 ± 40.4	0.036
ALT, IU/L	82.7 ± 24.4	121 ± 25.6	0.283
ALP, IU/L	327 ± 38.8	439 ± 40.6	0.052
γ-GTP, IU/L	95.3 ± 36.6	240 ± 38.3	0.008

*CRP* C-reactive protein, *AST* Aspartate aminotransferase, *ALT* Alanine aminotransferase, *ALP* Alkaline phosphatase, *γ-GTP* Gamma-glutamyl transpeptidase

Continuous variables are presented as mean ± SEM

**Table 4:** Comparison of perioperative characteristics according to the difficulty of cholecystitis

Variable	Group A	Group B	*P* Value
	(*n* = 35)	(*n* = 32)	
Operating time, min	146 ± 5.8	97.3 ± 6.1	<0.0001
Blood loss, mL	180 ± 32.1	28.8 ± 33.6	0.0017
Laparoscopic cholecystectomy, n (%)	21 (60.0)	24 (75.0)	0.192
Open cholecystectomy, n (%)	5 (14.3)	8 (25.0)	0.268
Open conversion, n (%)	9 (25.7)	0 (0)	0.002
Subtotal cholecystectomy and/or mucoclasis, n (%)	25 (71.4)	0 (0)	<0.0001
Necrosis and/or abscess, n (%)	24 (68.6)	0 (0)	<0.0001
Tight adhesion at the gallbladder neck, n (%)	20 (57.1)	0 (0)	<0.0001
Gallbladder stone, n (%)	30 (85.7)	28 (87.5)	0.831

Continuous variables are presented as mean ± SEM

**Fig. 2:**
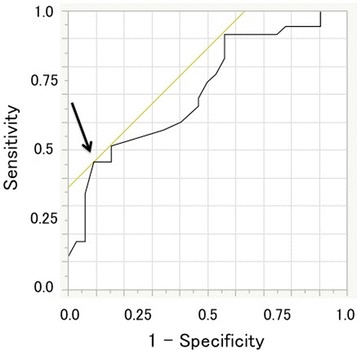
ROC curve analysis of the interval between PTGBD and cholecystectomy and the technical difficulty of cholecystectomy. The area under the curve (AUC) is 0.712. The cut-off value for the interval between PTGBD and cholecystectomy was calculated as 216 h

ROC curve analysis yielded a value of 216 h as the optimal cut-off interval between PTGBD and cholecystectomy, in relation to the difficulty of cholecystectomy (Fig. 2). Using this cut-off value, patients were divided into the short interval (SI) (*n* = 14) and the long interval (LI) (*n* = 53) groups. The perioperative outcomes are shown in Table 5. The number of patients who underwent difficult cholecystectomy was significantly greater in the SI group than in the LI group (85.7 vs. 43.4%; *p* = 0.005). Compared with the LI group, the SI group had significantly longer operating time (143 ± 10.9 min vs. 117 ± 5.6 min; *p* = 0.05); higher rates of necrosis and/or abscess formation (66.7 vs. 30.2%; *p* = 0.016) and adhesions around the gallbladder neck (50.0 vs. 22.6%; *p* = 0.043); and higher incidence of postoperative complications, except surgical site infection (35.7 vs. 7.6%; *p* = 0.006). In particular, intraabdominal abscess developed more frequently in the SI group than in the LI group (21.4 vs. 0%; *p* = 0.006). Duration of postoperative hospital stay did not differ between the two groups (SI group vs. LI group, 10.6 ± 1.6 days vs. 8.6 ± 0.81 days; *p* = 0.269).

**Table 5:** Comparison of perioperative characteristics and postoperative outcomes according to the interval between PTGBD and cholecystectomy

Variable	SI group	LI group	*P* value
	*n* = 14	*n* = 53	
Operating time, min	143 ± 10.9	117 ± 5.6	0.041
Blood loss, mL	199 ± 53.2	83.8 ± 27.3	0.057
Difficult cholecystectomy, n (%)	12 (85.7)	23 (43.4)	0.005
Open conversion, n (%)	2 (15.4)	7 (17.1)	0.887
Subtotal cholecystectomy and/or mucoclasis, n (%)	6 (42.9)	19 (35.9)	0.630
Necrosis and/or abscess, n (%)	8 (66.7)	16 (30.2)	0.016
Tight adhesion at the gallbladder neck, n (%)	7 (50.0)	13 (24.5)	0.064
Postoperative complications except SSI, n (%)			0.006
Yes	5 (35.7)	4 (7.6)	
No	9 (64.3)	49 (92.4)	
Bile leak, n (%)	0 (0)	1 (1.9)	0.605
Intraabdominal abscess, n (%)	3 (21.4)	0 (0)	0.0006
Hematoma, n (%)	0 (0)	1 (1.9)	0.605
Paralytic ileus, n (%)	1 (7.1)	2 (3.8)	0.588
SSI, n (%)	1 (7.1)	3 (5.7)	0.835
Postoperative hospital stay, days	10.6 ± 1.6	8.6 ± 0.81	0.269

*SI group* Short interval group, *LI group* Long interval group, *SSI* Surgical site infection

Continuous variables are presented as mean ± SEM

## Discussion

Recently, immediate LC has become standard treatment for AC [7, 8]. However, we sometimes experience difficulty during LC in AC patients with severe local inflammation; this can increase the rate of postoperative complications, such as bile leak, common bile duct injury, and bowel injury [2]. Furthermore, some investigators reported that the mortality rate was as high as 18–50% in elderly patients or those with severe comorbidities [9–11]. On the other hand, PTGBD is not a difficult procedure with a very low mortality rate [12]. Therefore, PTGBD has been performed as a safer substitute for cholecystectomy in such high-risk patients [13, 14]. In this study, the rate of unsuccessful treatment using PTGBD was 3.9%, and this result was acceptable compared with that in a previous report.

The TG13 has indicated the severity criteria for AC and its appropriate management, including PTGBD [3, 4], but the optimal timing for subsequent cholecystectomy after PTGBD was not mentioned. Chikamori et al. suggested that early LC following PTGBD was safe and effective [15]. On the other hand, Kim et al. suggested that delayed LC following PTGBD decreased the rates of open conversion and complications [16]. Yamada et al. suggested that the interval between PTGBD and cholecystectomy was not related to the amount of blood loss, operating time, and open conversion rate [17]. Therefore, the optimal timing for subsequent cholecystectomy after PTGBD is still controversial.

In this study, grade III AC patients who have organ dysfunction were enrolled. Although organ dysfunction seemed to negatively affect postoperative morbidity and mortality, all of grade III AC patients underwent operation after complete recover from organ dysfunction. Therefore, organ dysfunction did not affect postoperative outcomes.

At the beginning of this retrospective study, we assessed whether the interval between PTGBD and cholecystectomy was a risk factor for a difficult surgery. Based on our results, the interval between PTGBD and cholecystectomy was short in patients who underwent difficult cholecystectomy. Comparison of outcomes according to a cut-off interval of 216 h showed a significantly higher rate of postoperative complications, especially intraabdominal abscess, and prolonged operating time in the SI group than the LI group. These results indicated that cholecystectomy should be considered at least 216 h after PTGBD.

The proper definition of difficult cholecystectomy is crucial in this study. Although conversion to open cholecystectomy reflects a difficult LC, it could also depend on the surgeon’s ability. In addition to conversion rate, objective and subjective criteria for difficult cholecystectomy were employed in this study based on results of previous reports. In particular, gallbladder inflammation was a risk factor for technical difficulty and conversion from LC to open cholecystectomy [18–20]. We believed that our criteria for difficult cholecystectomy were appropriate. Although BMI, CRP, and the interval between symptom onset and PTGBD were suggested as independent risk factors for difficult cholecystectomy [17–21], these parameters did not differ between difficult and non-difficult cholecystectomy cases in this study.

In this study, early (within 72 h) cholecystectomy following PTGBD was performed only in seven cases. Therefore, the usefulness of early cholecystectomy could not be adequately assessed. AC progresses from edematous cholecystitis through necrotizing cholecystitis to sub-acute cholecystitis in about 10 days, and edematous phase, which is a phase before proceeding tight adhesion, lasts for 72 h after symptom onset [22]. Necrotizing cholecystitis is also reported to be one of the risk factor for difficult LC [23, 24]. Thus, the Tokyo Guidelines recommend early LC. Accordingly, early cholecystectomy following PTGBD might reduce the incidence of postoperative complications. Some investigators reported rapid symptomatic improvement in more than 90% patients after cholecystectomy within 24 to 72 h of PTGBD [25–27]. Indeed, some patients can safely undergo cholecystectomy soon after PTGBD. However, early LC following PTGBD for grade III cholecystitis seems challenging because of the accompanying severe organ dysfunction. Therefore, in patients who do not recover from organ dysfunction within 72 h, we should not perform early LC.

One limitation of our study was inclusion of patients who underwent three different procedures (LC, conversion from LC to open cholecystectomy, and open cholecystectomy). Because operating time and blood loss could be confounded by these procedures, these two parameters had to be excluded from the criteria for difficult cholecystectomy. Surgical site infection was not included as a postoperative complication because of the same reason. The combination of three different procedures might also explain the same length of postoperative hospital stay among the patients, regardless of the interval between PTGBD and cholecystectomy.

In this study, the cut-off value of 216-h interval between PTGBD and cholecystectomy was similar with that of a previous report (7 days) [16]. In general, when inflammation occurs, cytokines are produced and they cause fibrosis in 7 days [28]. It is also reported that further fibrosis does not occur after 7 days [29]. Therefore, there is a possibility that waiting for longer time leads to a safer cholecystectomy. However, there is no evidence regarding this, and further examination is required.

## Conclusions

Cholecystectomy should be performed later than 216 h after PTGBD in patients with severe AC, although the possibility of a safe outcome of early LC within 72 h of PTGBD remains.

## Additional file

Additional file 1: Detailed data of patient characteristics used in this study. 1: yes, 0: no. (XLSX 49 kb)

## Abbreviations

AC: Acute cholecystitis
LC: Laparoscopic cholecystectomy
LI: Long interval
PTGBD: Percutaneous transhepatic gallbladder drainage
ROC: Receiver operating characteristic
SEM: Standard error mean
SI: Short interval
TG13: Tokyo Guidelines 2013

## References

[1] Yamashita Y, Takada T, Strasberg SM, Pitt HA, Gouma DJ, Garden OJ (2013). TG13 surgical management of acute cholecystitis. J Hepatobiliary Pancreat Sci.

[2] Lee SW, Yang SS, Chang CS, Yeh HJ (2009). Impact of the Tokyo guidelines on the management of patients with acute calculous cholecystitis. J Gastroenterol Hepatol.

[3] Sekimoto M, Takada T, Kawarada Y, Nimura Y, Yoshida M, Mayumi T, Miura F, Wada K, Hirota M, Yamashita Y, Strasberg S. Need for criteria for the diagnosis and severity assessment of acute cholangitis and cholecystitis: Tokyo guidelines. J Hepato-Biliary-Pancreat Surg 2007;14:11-14. 10.1007/s00534-006-1151-z PMC2784506 17252292

[4] Yokoe M, Takada T, Strasberg SM, Solomkin JS, Mayumi T, Gomi H (2012). New diagnostic criteria and severity assessment of acute cholecystitis in revised Tokyo guidelines. J Hepatobiliary Pancreat Sci.

[5] Radder RW (1980). Ultrasonically guided percutaneous catheter drainage for gallbladder empyema. Diagn Imaging.

[6] Habib FA, Kolachalam RB, Khilnani R, Preventza O, Mittal VK (2001). Role of laparoscopic cholecystectomy in the management of gangrenous cholecystitis. Am J Surg.

[7] Borzellino G, Sauerland S, Minicozzi AM, Verlato G, Di Pietrantonj C, de Manzoni G (2008). Laparoscopic cholecystectomy for severe acute cholecystitis. A meta-analysis of results. Surg Endosc.

[8] Gurusamy K, Samraj K, Gluud C, Wilson E, Davidson BR (2010). Meta-analysis of randomized controlled trials on the safety and effectiveness of early versus delayed laparoscopic cholecystectomy for acute cholecystitis. Br J Surg.

[9] Winbladh A, Gullstrand P, Svanvik J, Sandstrom P (2009). Systematic review of cholecystostomy as a treatment option in acute cholecystitis. HPB (Oxford).

[10] Morse BC, Smith JB, Lawdahl RB, Roettger RH (2010). Management of acute cholecystitis in critically ill patients: contemporary role for cholecystostomy and subsequent cholecystectomy. Am Surg.

[11] Laurila J, Syrjala H, Laurila PA, Saarnio J, Ala-Kokko TI (2004). Acute acalculous cholecystitis in critically ill patients. Acta Anaesthesiol Scand.

[12] Ito K, Fujita N, Noda Y, Kobayashi G, Kimura K, Sugawara T (2004). Percutaneous cholecystostomy versus gallbladder aspiration for acute cholecystitis: a prospective randomized controlled trial. AJR am J Roentgenol.

[13] Chopra S, Dodd GD, Mumbower AL, Chintapalli KN, Schwesinger WH, Sirinek KR (2001). Treatment of acute cholecystitis in non-critically ill patients at high surgical risk: comparison of clinical outcomes after gallbladder aspiration and after percutaneous cholecystostomy. AJR am J Roentgenol.

[14] Davis CA, Landercasper J, Gundersen LH, Lambert PJ (1999). Effective use of percutaneous cholecystostomy in high-risk surgical patients: techniques, tube management, and results. Arch Surg.

[15] Chikamori F, Kuniyoshi N, Shibuya S, Takase Y (2002). Early scheduled laparoscopic cholecystectomy following percutaneous transhepatic gallbladder drainage for patients with acute cholecystitis. Surg Endosc.

[16] Kim HO, Ho Son B, Yoo CH, Ho Shin J (2009). Impact of delayed laparoscopic cholecystectomy after percutaneous transhepatic gallbladder drainage for patients with complicated acute cholecystitis. Surg Laparosc Endosc Percutan Tech.

[17] Yamada K, Yamashita Y, Yamada T, Takeno S, Noritomi T (2015). Optimal timing for performing percutaneous transhepatic gallbladder drainage and subsequent cholecystectomy for better management of acute cholecystitis. J Hepatobiliary Pancreat Sci.

[18] Asai K, Watanabe M, Kusachi S, Matsukiyo H, Saito T, Kodama H (2014). Risk factors for conversion of laparoscopic cholecystectomy to open surgery associated with the severity characteristics according to the Tokyo guidelines. Surg Today.

[19] Hutchinson CH, Traverso LW, Lee FT (1994). Laparoscopic cholecystectomy. Do preoperative factors predict the need to convert to open?. Surg Endosc.

[20] Sippey M, Grzybowski M, Manwaring ML, Kasten KR, Chapman WH, Pofahl WE (2015). Acute cholecystitis: risk factors for conversion to an open procedure. J Surg res.

[21] Bickel A, Hoffman RS, Loberant N, Weiss M, Eitan A (2016). Timing of percutaneous cholecystostomy affects conversion rate of delayed laparoscopic cholecystectomy for severe acute cholecystitis. Surg Endosc.

[22] Brodsky A, Matter I, Sabo E, Cohen A, Abrahamson J, Eldar S (2000). Laparoscopic cholecystectomy for acute cholecystitis: can the need for conversion and the probability of complications be predicted? A prospective study. Surg Endosc.

[23] Hayama S, Ohtaka K, Shoji Y, Ichimura T, Fujita M, Senmaru N (2016). Risk factors for difficult laparoscopic cholecystectomy in acute cholecystitis. JSLS.

[24] Iwashita Y, Hibi T, Ohyama T, Honda G, Yoshida M, Miura F (2017). An opportunity in difficulty: Japan-Korea-Taiwan expert Delphi consensus on surgical difficulty during laparoscopic cholecystectomy. J Hepatobiliary Pancreat Sci.

[25] Han IW, Jang JY, Kang MJ, Lee KB, Lee SE, Kim SW (2012). Early versus delayed laparoscopic cholecystectomy after percutaneous transhepatic gallbladder drainage. J Hepatobiliary Pancreat Sci.

[26] Vogelzang RL, Nemcek AA (1988). Percutaneous cholecystostomy: diagnostic and therapeutic efficacy. Radiology.

[27] Patterson EJ, McLoughlin RF, Mathieson JR, Cooperberg PL, JK MF (1996). An alternative approach to acute cholecystitis. Percutaneous cholecystostomy and interval laparoscopic cholecystectomy. Surg Endosc.

[28] Holmdahl L, Ivarsson ML (1999). The role of cytokines, coagulation, and fibrinolysis in peritoneal tissue repair. Eur J Surg.

[29] Divilio LT (2005). Surgical adhesion development and prevention. Int Surg.

